# Functional Heart Disease

**Published:** 1910-03

**Authors:** Geo. F. Butler

**Affiliations:** Professor and head of the Department of Therapeutics and Professor of Preventive and Clinical Medicine, College of Medicine and Surgery (Medical Department Valparaiso University); Chicago, Ill.


For Texas Medical Journal.

### Functional Heart Disease.

BY GEO. F. BUTLER, M. D.,

Professor and head of the Department of Therapeutics and Professor of Preventive and Clinical Medicine, College of Medicine and Surgery (Medical Department Valparaiso University).

The diseases of the heart may be classified as functional diseases, structural diseases, inflammatory diseases and results. The principal functional disturbances of the heart are due to, first, palpitation; second, weak heart; third, angina pectoris; fourth, exophthalmic goiter. The first two are really the only strictly functional disorders of the heart, they are also termed cardiac neuroses.

*Palpitation of the Heart.*—Palpitation is a forcible, disturbed and rapid action of the heart. The principal causes of palpitation are nervousness, dyspepsia, so-called, rheumatism and hypertrophy. It is also a symptom of disease of this organ (e. g., inflammation) and also is met in connection with disease of remote organs. Nervous palpitation is encountered in anemic persons and especially in hysterical women. The hurry and worry of life may cause attacks of palpitation. Dyspeptic palpitation is met in coffee and tea lovers, tobacco users, spirit drinkers and high livers generally, also in those who bolt their food. Certain occupations which interfere with digestive functions also produce palpitation. Rheumatism develops palpitation by the fever, as well as by the lessened alkalinity of the blood.

This lessened alkalinity, easily demonstrated by simple test in the higher degree of urinary acidity, can readily be overcome by large doses of alkalies and saline elimination. As an alkali my choice is the following, devised sometime since and now marketed, for short, as sodoxylin: Each sixty grains (an average rounding teaspoonful) contains repurified sodium sulphocarbolate, grs. 2½; sodium sulphate, grs. 5; sodium bicarbonate, grs. 20; colchicine, gr. 1-500; juglandin, gr. 1-6; xanthoxylin, gr. 1-6; with aromatics. This is an eliminant, intestinal antiseptic and antacid of exceeding utility.

As an eliminant, in addition to the above, and at the same time a marked hepatic stimulant, I prefer the action of salithia 60 per cent magnesium sulphate in effervescent combination with lithium and colchicine.

We may know the disorder is functional and not structural when the palpitation is not increased by moderate exercise. If increased it may be inferred that it is due to hypertrophy with possible valvular lesion.

The alarming symptoms in a case of palpitation of the heart are dizziness, headache, trembling, choking and faintness. An outline of treatment includes, first, rest in recumbent posture; second, diversion of mind; third, a drink of water or something warm, and, fourth, cactin until effect.

*Weak Heart.*—The chief symptoms of weak or irritable heart are faintness, vertigo on extra exertion or excitement. There may also be headache with cardiac pain or distress. The chief causes of weak heart are overexertion, overwork and mental excitement. The real pathological conditions may be spinal or nerve center hyperemia, unless dependent upon local diseases elsewhere. Weak heart is an inheritance of old age. The outline of management of a case of weak heart depends something upon the history. Rest is of the first importance. A supporting diet must be carefully selected. The mind should be diverted, a country or seaside resort should be selected. A visit to the mountains or a European trip should be discountenanced. A diffusive stimulant should be at hand to ward off attacks of heart failure, glonoin being the best, and strychnine and digitalin should be used supportively to effect.

*An Exophthalmic Heart.*—Rapid action of the heart with enlarged thyroid, prominent eyes are diagnostic signs of exophthalmic goiter. This disease may be classed as a neurosis of the cardiac plexus with the sympathetic and its connection. This disease is usually slow in making its appearance. It may be remittant, as during and after pregnancy, and may disappear, but unless arrested it usually grows worse. It produces or is attended with rapid action of the heart. The prognosis should be guarded. While it may disappear, the usual course is to produce hypertrophy and dilatation of the heart with valvular insufficiency, aneurism of the thyroidal vessels, an inflammation and ulceration of the cornea of the eyes.

The supposed causes of this disease are cardiac neuroses, mechanical obstruction in the gland, mental excitement and calcareous water.

*Treatment and Remedies.*—The indications are: Remove the cause if possible, select a proper diet, control the life, apply galvanism to the thyroid, driving the blood away from the glands and cord. In the early stages with active hypertrophy the indicated remedy is veratrine, or aconitine if the stomach is irritable. Either should be given to the exact desirable effect. When the circulation begins to fail the less contractile cardiac tonics are often the most useful remedies, such as cactin and sparteine or strophanthin rather than digitalin. Thyroid extracts do harm. The serum from thyroidectonized goats has recently been applied and, it is claimed, with more or less beneficial effects. In other cases sodium salicylate, sodium glycerophosphate, quinine bromide and other agents have been found useful. In many cases we find that the triple arsenates of iron, quinine, strychnine with nuclein act admirably.

*Angina pectoris* or *stenia cordis* is an irregular, paroxysmal attack of cardiac pain with a feeling of impending death. It is supposed to be of nervous origin (spinal hyperemia), with or without cardiac lesions, e. g., degeneration of the coronary arteries, hypertrophy and valvular lesions. The attacks are ushered in suddenly with a feeling of constriction and severe pain at the heart as if held by a hand. The patient imagines he can not breathe, but gets a deep breath now and then. Sometimes the pain radiates down the left side. The extremities are cold, the face pale at first, but become finally flushed, with anxious expression as of impending death. The paroxysms are usually short, and where severe simulate epilepsy. Extra mental or physical exertion, or fright, may precipitate an attack. Men are more frequently sufferers from this disease than women. The pathology seems to be a spinal and cardiac hyperemia with, in some cases, cardiac lesions as hypertrophy, calcification of the coronary arteries, valvular obstruction or insufficiency, atheromatous arteries or fatty heart. The prognosis should be guarded. The attacks are dangerous, but the patient often lives for years in fear and alarm. The treatment of an attack is as follows: Lay the patient in a comfortable position, and while attendants are loosening the clothing chloroform may be applied while the indicated remedies are getting control of the disease. Hypodermic injections of hyoscine, morphine and cactin (my choice) act very well to arrest the attacks. Nitrite of amyl is often used and the continual use of some nitrite, nitrite of sodium, or nitroglycerine or erythrol tetronitrate seem to prevent a return of the attack. Between the attacks regulate the habits and diet of the patient, continuing supportive tonics, like the triple arsenates with nuclein mentioned, at intervals to prevent recurrence.

Chicago, Ill.

